# The anti-viral dynamin family member MxB participates in mitochondrial integrity

**DOI:** 10.1038/s41467-020-14727-w

**Published:** 2020-02-26

**Authors:** Hong Cao, E. W. Krueger, Jing Chen, Kristina Drizyte-Miller, Mary E. Schulz, Mark A. McNiven

**Affiliations:** 1 Department of Biochemistry and Molecular Biology, Mayo Clinic, 200 1st Street SW, Rochester, MN 55905 USA; 2 Center for Basic Research in Digestive Diseases, Division of Gastroenterology & Hepatology, Mayo Clinic, 200 1st Street SW, Rochester, MN 55905 USA; 3 Biochemistry and Molecular Biology Program, Mayo Clinic Graduate School of Biomedical Sciences, Mayo Clinic, 200 1st Street SW, Rochester, MN 55905 USA

**Keywords:** Proteins, Mitochondria

## Abstract

The membrane deforming dynamin family members MxA and MxB are large GTPases that convey resistance to a variety of infectious viruses. During viral infection, Mx proteins are known to show markedly increased expression via an interferon-responsive promoter to associate with nuclear pores. In this study we report that MxB is an inner mitochondrial membrane GTPase that plays an important role in the morphology and function of this organelle. Expression of mutant MxB or siRNA knockdown of MxB leads to fragmented mitochondria with disrupted inner membranes that are unable to maintain a proton gradient, while expelling their nucleoid-based genome into the cytoplasm. These findings implicate a dynamin family member in mitochondrial-based changes frequently observed during an interferon-based, anti-viral response.

## Abstract (web summary)

Mx proteins belong to the dynamin family of large GTPases and are highly induced by interferon in virally infected cells. The authors show that uninfected immune cells and hepatocytes also express MxB protein that associates with mitochondria to alter the morphology and genome of this organelle.

## Introduction

Mitochondria are dynamic organelles that undergo fission and fusion events in response to changes in metabolic needs and important cellular processes such as cell division and apoptosis^1–3^. Central to these alterations in mitochondrial form are the dynamin family of large GTPases, which are known to bind and remodel membranes^4,5^. Distinct from the conventional dynamins (Dyn1, 2, 3) that are known to support membrane scission at distinct cellular sites such as the plasma membrane^6^, the Golgi apparatus^7^, as well as endosomes^8^ and autolysosomes^9–11^, the dynamin-related proteins have been implicated in the dynamics of both mitochondria^12^ and peroxisomes^13^. Although highly conserved within the N-terminal GTPase domains, these dynamin-related proteins are only modestly conserved in the middle domain and generally lack pleckstrin binding and proline-rich domains found in the conventional family members. The mitofusins (MFN1/MFN2) are believed to support the outer mitochondrial membrane fusion and optic atrophy 1 (OPA1) has been implicated in the fusion of the inner membrane^14–17^. Reciprocally, dynamin-related protein 1 (DRP1) has been shown to constrict the outer mitochondrial membrane^4^ with the final scission event perhaps driven by conventional Dyn2^18^.

In addition to the mitochondria-associated dynamins are the myxovirus resistance GTPases (MX1/2 human and MxA/B mouse), which are <40% similar to the conventional dynamins but over 60% identical in sequence to each other. These related proteins are expressed in cells upon exposure to type 1 interferon (IFN) and convey a cellular innate immune response to a variety of different pathogenic viruses^19–24^ including influenza, hepatitis B (MxA), and HIV-1 (MxB). We and others have found that MxA can assemble into polymers that act to deform membranes^25,26^ and appears to reside on components of the smooth endoplasmic reticulum (ER)^25,27^. MxB also assembles into polymeric structures^28^ and was originally found to associate with nuclear pores where it was predicted to restrict the access of a viral genome to the host transcriptional machinery^29^. MxB has been described to bind to the HIV-1 genome, while impairing its chromosomal integration^24,30,31^, and recently has been identified as a key factor behind IFN-mediated suppression of hepatitis C virus infection^32^.

In this study we report that although markedly upregulated in cells treated with IFN, MxB is expressed constitutively in a variety of cell types, particularly in primary hepatocytes and hepatoma cell lines. In addition to reported localization at nuclear pores, we find that MxB is intimately associated with the inner mitochondria, where it plays an essential role in the maintenance of mitochondrial cristae and DNA stability of this organelle.

## Results

### MxB associates with mitochondria in cultured cells

**Fig. 1:**
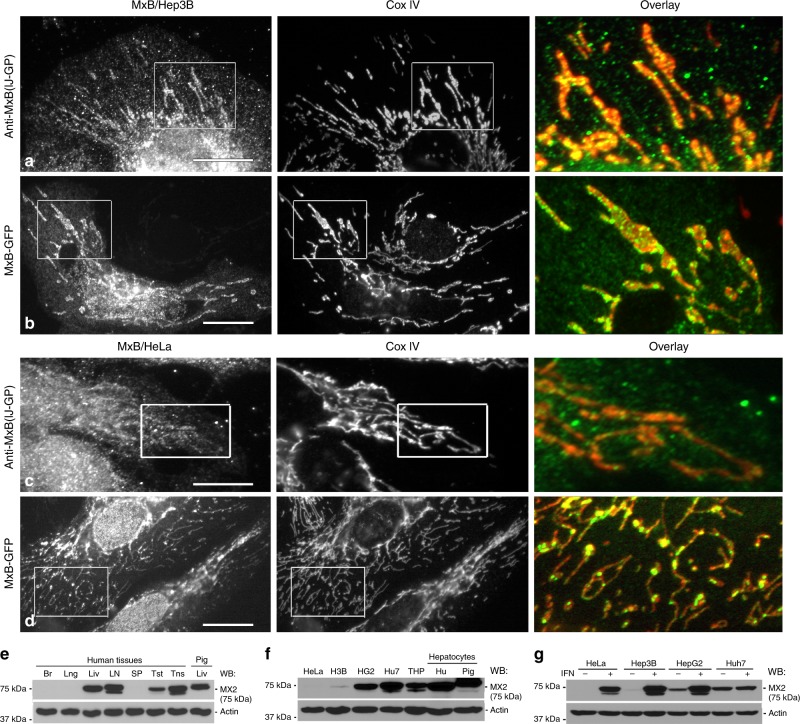
MxB is localized to mitochondria in cultured cells. Fluorescence images of Hep3B cells or HeLa cells, stained with a polyclonal antibody (IJ-GP) to MxB (**a**, **c**), or expressing exogenous human MxB-GFP (**b**, **d**). All cells were stained for the inner mitochondrial membrane marker CoxIV. MxB is visualized as small or large puncta that align closely with mitochondrial membranes. Boxed regions provide a higher magnification for color overlays showing the alignment of MxB (green) with mitochondria (red) in both cell types. Scale bars, 10 μm. **e** Western blot analysis of different human tissues (Br = brain, Lng = lung, Liv = liver, LN = lymph node, SP = spleen, Tst = testis, Tns = tonsil) for endogenous MxB that appears enriched in the liver, lymph node, testis, tonsil, and pig liver. **f** Western blotting comparing MxB expression in HeLa cells, three different hepatocyte cell lines, and a human monocyte cell line (THP-1), as well as primary human and pig hepatocytes. Expression levels are exceptionally high in primary hepatocytes, the HepG2 and Huh7 cell lines (H3B = Hep3B, HG2 = HepG2, Hu7 = Huh7, THP = THP-1, Hu = human hepatocytes). **g** Western blotting of four distinct cell lines comparing endogenous MxB expression under control conditions vs. treatment with 1,000 IU/ ml^-1^ of IFN-α−2Α. HeLa, Hep3B and HepG2 cells induced MxB expression by IFN treatment, but not Huh7 cells.

As the related family member MxA has been shown to convey a partial resistance to the hepatocyte-centric HBV^33^, while associating with membranous structures in hepatocytes, our goal is to compare the distribution and expression of the highly related family member MxB in hepatocyte cell lines and in the whole liver. Using several different antibodies and tagged constructs to MxB, we observe the punctate nuclear staining previously observed by others^34–36^. In addition to this localization, we observe a fusiform and linear staining pattern in the hepatoma cell line Hep3B and HeLa cells, using a previously published MxB antibody and exogenously expressed green fluorescent protein (GFP)/Myc-tagged constructs (Fig. 1a–d and Supplementary Fig. 1a–c). Co-staining these cells with a variety of different organelle marker antibodies reveals that the MxB labeling represents mitochondria as confirmed by antibodies to cytochrome c oxidase 4 (CoxIV) (Fig. 1a–d and Supplementary Fig. 1a–c). This localization is consistent when staining with three distinct MxB antibodies or expressing either GFP or Myc-tagged MxB, whereas the characteristics of labeling using these different probes varies from fine to large puncta coating the mitochondria.

Western blot (WB) analysis of various human tissues for endogenous MxB shows that MxB appears enriched in the liver, lymph node, testis, tonsil, and pig liver. Of note is the fact that human lymph node and tonsil tissues possess two bands, whereas the liver, testis, and pig liver indicate a single band (Fig. 1e). Accordingly, appreciable levels of endogenous MxB are observed in several human hepatoma cell lines (Hep3B, HepG2, Huh7) and primary hepatocytes isolated from human and pig livers (Fig. 1f). HeLa cells appear to have modest MxB levels that become more apparent upon longer exposures (Supplementary Fig. 4). As reported previously^34^, MxB expression is markedly increased in isolated cells treated with 1000 IU ml^−1^ of IFNα (Fig. 1g).

MxB has been shown to be expressed in two forms, one of which contains an N-terminal sequence required for nuclear targeting. The distribution of the long and short MxB forms have been examined in significant detail by others^33,34^. In our hands we find that the short form has modest affinity for the nucleus or mitochondria regardless of the tag used in comparison with the long form that associates with both organelles (Supplementary Fig. 1d-j).

**Fig. 2:**
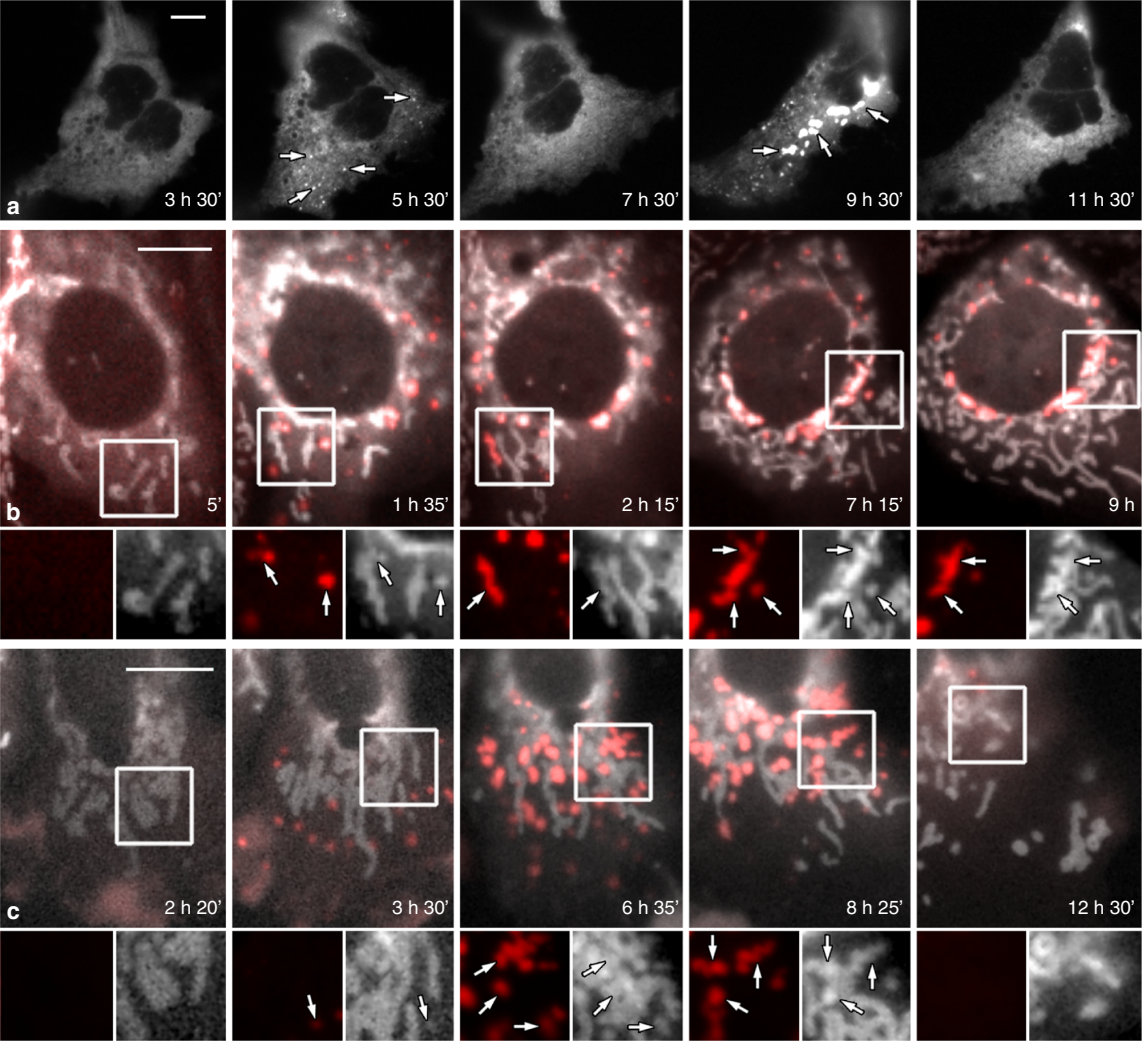
MxB distribution in living cells is dynamic. **a** Time-lapse imaging of Hep3B cells expressing mCherry-MxB and viewed over a 16 h time period. Initially, MxB is diffuse in distribution, although condenses into several fine puncta by 5 h that subsequently dissolve but reform into much larger structures by 9 h. Surprisingly, these large aggregates also dissolve or disassemble several hours later (see Supplementary Movie 1). **b**, **c** Time-lapse imaging of Hep3B cells co-expressing mCherry-MxB (red) and Mito-GFP (white), and viewed over a 16 h time period to test for dynamic associations of MxB with mitochondria. Low-magnification images of cells show the tagged MxB changing from a diffuse to punctate, to diffuse distribution in close proximity of mitochondria. Higher magnification viewing (inserts) reveals that many of these formed puncta associate along or at the tips of the dynamic mitochondria (arrows) (see Supplementary Movies 2 and 3). Scale bars, 10 μm.

As our initial morphological observations (Fig. 1 and Supplementary Fig. 1) show MxB associating with mitochondria as either small or large puncta, or more diffuse in nature, we think it is important to analyze live cells expressing MxB-mCherry to test for changes in MxB localization over time. One example of this dynamic distribution is depicted in Fig. 2a, showing a single Hep3B cell expressing MxB-mCherry viewed over a 16 h time period. Dramatic and unexpected changes in MxB localization are observed, as the tagged protein appears to cycle between diffuse and punctate forms during the extended viewing period. Subsequently, cells co-transfected with Mito-GFP^37^ as a mitochondrial marker test whether the coalescing MxB puncta might associate with this organelle. Indeed, as shown in the images from movies of two distinct live cells (Fig. 2b, c), MxB-mCherry appears diffuse and barely detectable at early viewing times but subsequently coalesces into numerous large puncta that associate with the tips of undulating mitochondria (inserts). As observed in Fig. 2a, these puncta appear to dissolve over time, leaving the MxB in a diffuse state.

### MxB manipulations disrupt mitochondrial structure and function

**Fig. 3:**
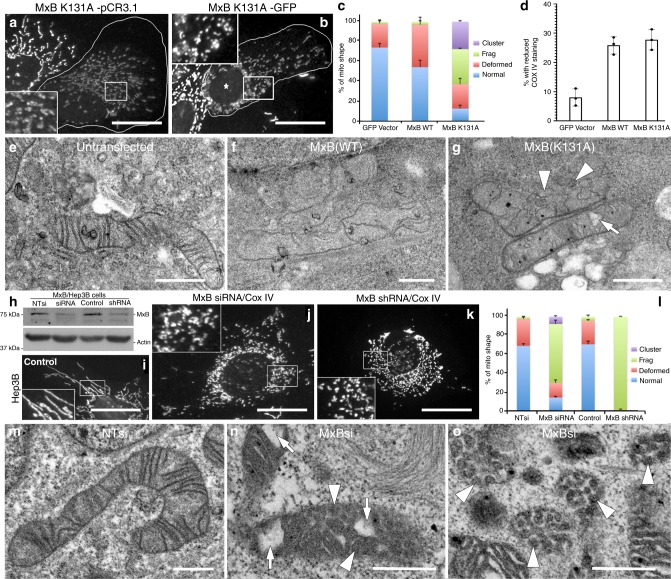
Manipulation of MxB expression induces marked changes in mitochondrial morphology. **a**, **b** Fluorescence micrographs of transfected Hep3B cells (outlined in white border) expressing either an untagged (**a**) or GFP-tagged (**b**) GTPase-defective K131A mutant of MxB. Transfected cells possess mitochondria that are fragmented or distorted, or exhibit attenuated staining with antibodies to CoxIV compared with adjacent untransfected cells. **c** Bar graph representing morphological changes of mitochondria in control, WT, or mutant (K131A) MxB-expressing cells. Morphologies of clustered, fragmented, deformed, or normal mitochondria were counted in images of 636 Hep3B cells from three distinct experiments. **d** Graph representing the mean percentage of cells exhibiting a reduced CoxIV staining from 636 control or MxB-transfected cells from three distinct experiments. **e**–**g** Electron micrographs of mitochondria from Hep3B cells transfected to express MxB WT or K131A. Control cells (**e**) possess mitochondria with normal cristae, while cells expressing WT (**f**) or mutant (K131A) MxB (**g**) display mitochondria with reduced cristae and dark membrane vesiculations in the matrix (arrowheads). Scale bars, 10 μm (**a**, **b**), 0.5 μm (**e**–**g**). **h**–**o** Reducing MxB levels by siRNA or shRNA treatment promotes mitochondrial fragmentation**. h** Western blot of Hep3B cells treated for 5 days with siRNA or shRNA to MxB shows reduced protein levels. **i**–**k** Immunofluorescence of control Hep3B cells (**i**) or cells treated with siRNA (**j**) or shRNA (**k**) to reduce endogenous MxB levels and stained for CoxIV. Both treatments induced a marked change in mitochondrial morphology. **l** Bar graph representing morphological changes of mitochondria from 568 Hep3B cells from three distinct experiments. **m**–**o** Electron microscopy of control NTsi (**m**) or siRNA MxB-treated cells showing fragmented mitochondria with vesiculated cristae (arrowheads) and large vacuoles (arrows). (**c**, **d**, **l** Data were analyzed using a two-tailed *t*-test, error bars indicate SD. Scale bars, 10 μm (**i**–**k**), 0.5 μm (**m**–**o**).

Based on the morphological observations described above, we next test whether manipulation of MxB levels might alter mitochondrial morphology. Hep3B cells transfected with either GFP-tagged or untagged wild-type (WT) or GTPase-defective mutant K131A MxB for 24 h are followed by staining with the CoxIV marker antibody. Representative images displayed in Fig. 3a, b show that mutant MxB-transfected cells possess fragmented mitochondria and significantly reduced CoxIV staining compared with adjacent untransfected cells. Importantly, in many transfected cells, mitochondrial distribution, size, and shape appear abnormal. These altered phenotypes include fragmented, twisted, or clustered morphologies (Fig. 3c and Supplementary Fig. 3a). It is particularly surprising that 25–30% of the cells examined have markedly attenuated CoxIV staining (Fig. 3d). To define these changes at a higher resolution, Hep3B cells transfected to express WT or K131A mutant MxB for 2 days are then fixed and embedded for electron microscopy (EM). Although untransfected cells display normal mitochondrial morphologies, many Hep3B cells expressing either of the constructs possess mitochondria with a greatly reduced number of cristae and/or numerous vacuoles or blisters (Fig. 3e–g). Similar results are seen in transfected HeLa cells, which lose almost all of their mitochondrial cristae, leaving hollow, elongated, organelle shells (Supplementary Fig. 2a–c). To better understand the effects of the mutant K131A form, we compare the distribution of this mutant with the WT form in Hep3B cells that are transfected with GFP-tagged constructs then fixed and viewed by fluorescence microscopy. Quantification of mitochondrial and nuclear fluorescence using ImageJ software shows a near-equal preference of the WT form for both organelles, while the K131A mutant exhibits increased association with mitochondria (Supplementary Fig. 2d). As a biochemical comparison, Hep3B cells expressing either the WT or K131A form subjected to subcellular fractionation to obtain nuclear, mitochondrial enriched, and cytosol fractions, are blotted for corresponding organelle markers and MxB (Supplementary Fig. 2e). By this approach, a near-equal distribution of both forms is observed.

To further define the role of MxB in maintaining mitochondrial morphology, Hep3B cells are subjected to MxB knockdown using both small interfering RNA (siRNA) and short hairpin RNA (shRNA) approaches. Following 5 days of treatment, cells are fixed and stained for CoxIV (Fig. 3h–k) or prepared for EM (Fig. 3m–o). As observed in cells expressing exogenous WT or mutant (K131A) MxB protein, these knockdown cells display altered mitochondrial morphologies including fragmented, twisted, or clustered shapes. A comprehensive table showing the effects of MxB knockdown, or overexpression, on mitochondrial shape is provided in Supplementary Fig. 3a. In contrast to the cells overexpressing tagged MxB proteins, the knockdown cells did not exhibit reduced staining for CoxIV or Tom20. EM of siRNA-treated Hep3B cells, however, did reveal highly altered mitochondria with deformed shapes, large vacuoles, and vesiculated cristae (Fig. 3m–o and Supplementary Fig. 2f–h). Based on our findings from the expression of either WT or mutant MxB proteins, and two distinct knockdown strategies, we conclude that this dynamin family member plays an important role in the maintenance of mitochondrial shape and integrity.

A central property of the Mx proteins is a substantially increased expression induced by IFN. Although MxB, but not MxA, is constitutively expressed in some cell and tissue types examined (Fig. 1e–g), the expression levels of both Mx proteins is markedly increased in cells treated with IFN (Fig. 1g, Supplementary Fig. 3j). We found that Hep3B cells treated with IFN for 48 h possess fragmented mitochondria in comparison with control cells (Supplementary Fig. 3b–d, h) and slightly increased levels of MxB on the nuclear envelope vs. mitochondria by immunostaining (Supplementary Fig. 3e-g,i). As a biochemical comparison, Hep3B cells treated as above with IFN for 48 h prior to lysis and differential centrifugation provides a rudimentary separation of nuclei from the mitochondria and cytosol. These fractions are run on SDS-polyacrylamide gel electrophoresis (PAGE) and probed by WB with antibodies to MxB and MxA (Supplementary Fig. 3j). This blotting is consistent with the cell staining and suggests that although there is a large increase in MxB associated with both organelles, a slight preference is observed with the nuclear fraction. As a control comparison the IFN -induced MxA protein is observed exclusively in the cytosolic fraction.

**Fig. 4:**
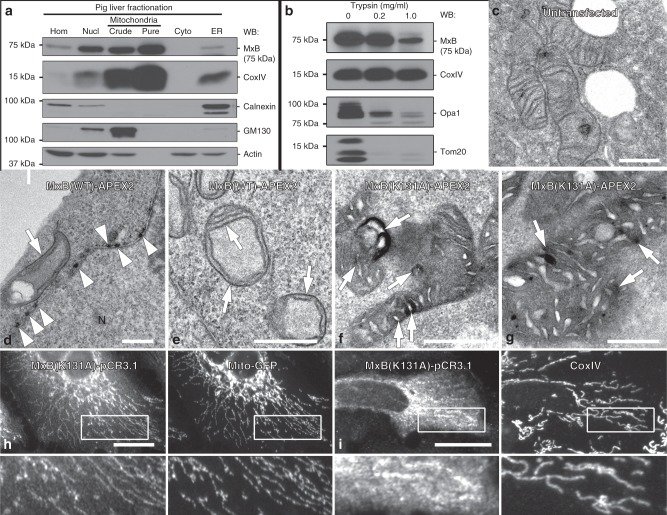
MxB resides within the mitochondrial matrix and is essential for normal mitochondrial function. **a** Western blot analysis of MxB distribution observed by subsequent steps of biochemical mitochondrial enrichment from pig liver. Fractions include: homogenate (Hom), a nuclear pellet (Nucl), a crude mitochondria fraction (Crude), a pure mitochondria fraction (pure), a cytosolic fraction (Cyto), and the endoplasmic reticulum (ER); all fractions were probed with marker antibodies to the mitochondria (CoxIV), actin, endoplasmic reticulum (Calnexin), and Golgi (GM130). Equal protein loads were used for each lane. **b** Western blot analysis of a mitochondria protease protection assay. A freshly isolated, enriched, mitochondria fraction from pig livers was treated with 0, 0.2, or 1 mg ml^−1^ of trypsin for 20 min, then probed with antibodies to MxB, or the mitochondrial proteins OPA1, Tom20, and CoxIV. MxB and respiratory complex enzyme CoxIV appear resistant to the higher levels (1.0 mg ml^−1^) of trypsin, whereas Tom20 and OPA1 are digested. **c**–**g** APEX2-based EM of the mitochondria in Hep3B cells. EMs of Hep3B cells exposed to transfection reagent as a control (**c**) or transfected to express either WT MxB (**d**, **e**) or mutant (K131A) MxB (**f**, **g**) fused to APEX2. Mitochondria in control cells show normal staining and structure, whereas WT MxB-expressing cells (**d**, **e**) exhibit prominent labeling of nuclear pores (arrow heads) and mitochondrial membranes (arrows). Mitochondrial cristae staining also appear reduced in these organelles. In contrast, mitochondria of MxB mutant-expressing cells (**f**, **g**) are darkly labeled throughout with exceptionally dark staining regions observed along the cristae (arrows) and in the matrix. These organelles also display vacuoles, protrusions, and disordered cristae. **h** HeLa cells and Hep3B cells (**i**) were co-transfected to express the mutant MxB K131A protein and a Mito-GFP marker or co-stained for CoxIV. Cells were fixed and stained for MxB. Both cell types showed an increased colocalization of the mutant MxB with mitochondria compared with WT protein. Scale bars, 0.5 μm (**c**–**g**), 10 μm (**h**, **i**).

As light microscopy imaging of MxB in cultured cells (Fig. 1) suggests an intra-mitochondrial localization consistent with the structural defects we observe by EM (Fig. 3), we utilize biochemical approaches to further define MxB localization. To this end, standard subcellular fractionation methods using pig liver as a tissue source^38,39^ are used. As displayed in Fig. 4a, a substantial enrichment of CoxIV is observed through sequential gradient centrifugation steps when comparing liver homogenate with a highly enriched mitochondrial fraction (Pure-Mito). Importantly, MxB protein levels are markedly increased in the “pure” mitochondrial fraction compared with all other isolated fractions, including the nuclear pellet fraction in which MxB is expected to reside. We next subject this “pure” mitochondrial preparation to a protease protection assay that is used often to define the topology of proteins associated with membranous organelles^40^. A freshly isolated, enriched, mitochondria fraction is treated with different concentrations of trypsin for 20 min at 4 °C then solubilized in sample buffer prior to SDS-PAGE and WB analysis for MxB and the mitochondrial proteins OPA1, Tom20, or CoxIV (Fig. 4b). Each of these four proteins display distinct sensitivities to the protease with the outer mitochondrial transmembrane protein Tom20 showing the most degradation followed by OPA1. MxB appears to be largely protected from the protease treatment while the inner membrane protein CoxIV remains intact, perhaps due to a tight association with the 12 other members of the respiratory Complex IV.

To support the morphological and biochemical observations described above, we attempt to define the localization of MxB at the ultrastructural level using a recently developed APEX2 approach for EM, in which cultured cells express the protein of interest tagged with a 28 kDa ascorbate peroxidase (APEX2)^41^. The chimeric protein maintains its activity within cells despite aldehyde fixation and allows the focused deposition of an electron-dense reaction product where the protein is localized. This application has been used to provide accurate localization for a series of known cellular proteins including several to different parts of the mitochondria including the outer mitochondrial membrane, the intermembrane space, and the matrix^41^. Accordingly, Hep3B cells expressing an MxB-APEX2 construct are then processed and viewed by EM. As shown in Fig. 4c, mock-transfected cells that are subsequently treated the same as MxB-APEX2-transfected cells, possess normal mitochondria of low contrast due to the lack of post staining. In contrast, cells expressing MxB WT-APEX2 exhibit dense staining of nuclear pores (Fig. 4d, arrowheads), consistent with the findings of others^41^ along with darkly stained outer and inner mitochondrial membranes (arrows). Many of these mitochondria also appear to a have reduced number of cristae. Cells expressing the mutant MxB K131A-APEX2 display mitochondria filled with considerably darker reaction product in the matrix (Fig. 4f, g), which is also observed in cells viewed by fluorescence microscopy (Fig. 4h,i).

**Fig. 5:**
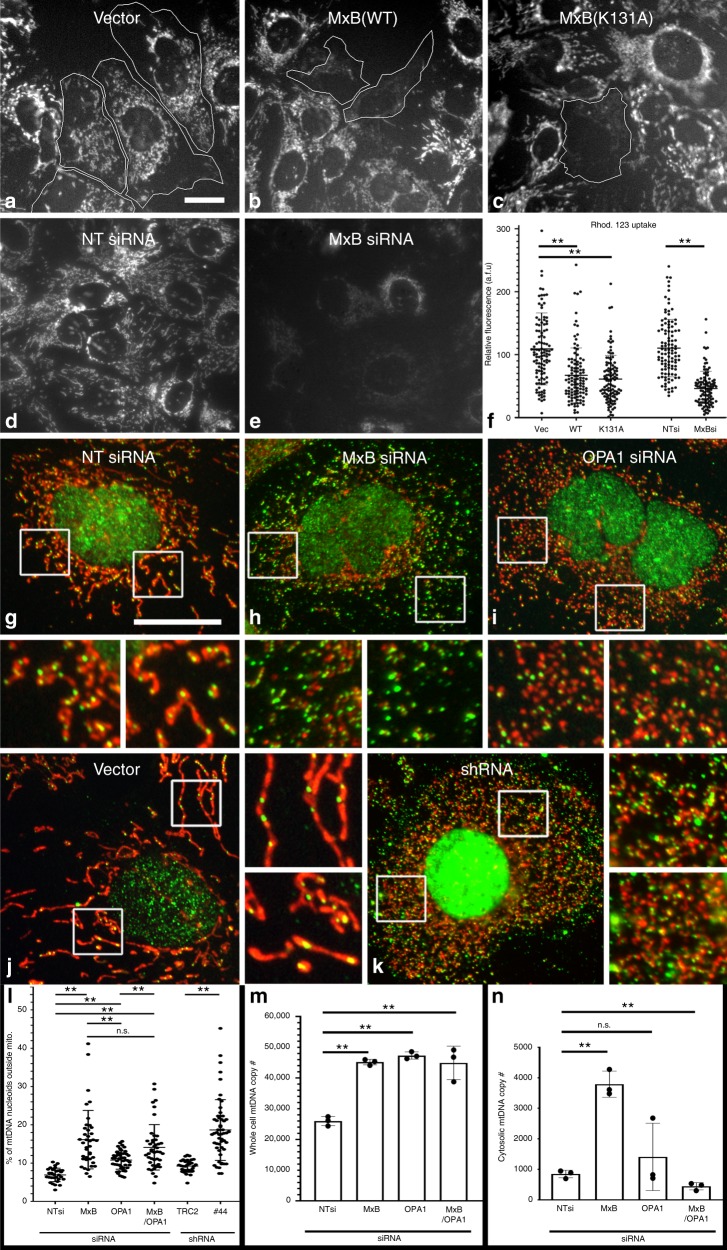
Expression of MxB disrupts mitochondrial function and genome stability. **a**–**f** Mitochondria in MxB-expressing cells exhibit loss of the proton gradient. Fluorescence micrographs of Hep3B cells that were transfected to express either pmCherry-N1 Vector, MxB WT-pmCherry, the MxB-pmCherry K131A mutant, or exposed to siRNA treatment for 5 days followed by incubation with 50 nM of the proton-gradient sensing dye Rho123. Transfected cells (white outlines) show modest mitochondrial fluorescence, indicating a metabolic impairment. **f** Graph quantification of the effects of MxB expression or siRNA knockdown on mitochondrial function (*n* = 529 cells over 3 independent experiments). **g**–**n** Release of the mitochondrial genome (mtDNA) into the cytoplasm in MxB-manipulated cells. Fluorescence micrographs of Hep3B cells treated for 5 days with control vectors and either siRNA or shRNA to reduce endogenous levels of MxB, OPA1, or both MxB and OPA1. Cells were then fixed and stained for mitochondrial DNA nucleoids (green) and CoxIV (red). Each manipulation shows low magnifications of representative cells with corresponding high-magnification images of boxed regions. Control-treated cells (**g**, **j**) exhibited close alignment of DNA with mitochondria, whereas siRNA- or shRNA- treated cells resulted in fragmented mitochondria and a significant number of DNA puncta residing in the cytoplasm (**h**, **k**). In comparison, OPA1 KD cells displayed shorter mitochondria with DNA nucleoids at the tips (**i**). **l** Fluorescence quantification of mtDNA loss into the cytoplasm under different treatment conditions. The *Y*-axis reflects relative amount of mtDNA outside the mitochondria (*n* = 244 microscopic fields over 4 independent experiments). **m**, **n** Graphs depicting quantitative PCR of either total cellular mtDNA (**m**) or cytosolic (**n**) mtDNA. Mean fold change in mtDNA copy number between NTsi, MxB, OPA1, and MxB/ OPA1 siRNA-treated cells was measured by qPCR using a mitochondrial D-loop primer set (**m**, **n**; *n* = 3 independent experiments). Data were analyzed using a two-tailed *t*-test, error bars represent SD. **l** NTsi vs. MxBsi, OPA1si, MxBsi /OPA1si, TRC2 vs. shRNA#44: all *P* < 0.0001, OPA1si vs. MxBsi/OPA1si: *P* = 0.0007; NS, not significant (*P* = 0.1827). **m** NTsi vs. MxBsi: *P* = 0.00082, OPA1si: *P* = 0.00069, MxB/OPA1si: *P* = 0.00023. **n** NTsi vs. MxBsi: *P* = 0.0027, OPA1si: NS (*P* = 0.2334), MxB/OPA1si *P* = 0.00305. Scale bars, 10 μm.

As the biochemical and morphological localizations of MxB are consistent with the observed changes in mitochondrial cristae in MxB-altered cells, it is important to test whether mitochondria within these cells had normal or impaired function. To this end, Hep3B cells are transfected to express vector, WT, or mutant MxB protein (Fig. 5a–c) or, alternatively, are treated with either non-targeted siRNA or siRNA to MxB for 5 days (Fig. 5d, e) prior to incubation with 50 nM Rho123 dye for 30 min as a probe for mitochondrial membrane potential. The capacity of the mitochondria to actively sequester this dye is widely used to measure the bioenergetics of this organelle^42^. Importantly, all cells in which MxB levels are manipulated show substantially less Rho123 fluorescence than did control cells (Fig. 5f), suggesting that cells with morphologically altered mitochondria due to MxB manipulation have reduced cristae and are functionally compromised.

### Loss of MxB expells mitochondrial genome into the cytoplasm

From the combined observations detailed above showing altered mitochondrial function, form, and disrupted cristae upon manipulations of MxB levels, we predict that these mitochondria might also exhibit alterations in their genome stability. Indeed, the mammalian dynamin family member OPA1 is also named MGM1 in yeast (for mitochondria genome maintenance), as the loss of this gene leads to disorganized cristae and loss of mitochondrial DNA^43,44^. The mitochondrial genome of 37 genes is packaged into spherical, punctate structures known as nucleoids, which can be observed using various DNA or protein antibodies^44–46^. To test the effects of MxB on the morphology and distribution of mitochondrial nucleoids, Hep3B cells are treated with siRNA or shRNA to MxB, as well as non-targeted siRNA or vector-alone shRNA (TRC2) as a control for 5 days prior to fixation and staining with antibodies to mitochondrial DNA (mtDNA) and CoxIV (Fig. 5g–k). OPA1 levels also reduced via siRNA are used as a comparison (Fig. 5i). In control cells, each mitochondria contains one to ten nucleoids (Fig. 5g, j). In striking contrast, the mitochondria of MxB knockdown cells are markedly fragmented (Fig. 5h, k) with fewer nucleoids and many of the nucleoids appear to reside within the cytoplasm. Morphological quantification of these cells shows a three to four fold increase in cytoplasmic distribution of mitochondrial nucleoids in MxB-knockdown cells vs. control cells (Fig. 5l). This loss of mtDNA is substantially less pronounced in cells subjected to a knockdown of the inner mitochondrial dynamin OPA1 (Fig. 5i, l). To support these morphological observations, we utilize quantitative PCR (qPCR) methods to measure total mtDNA as well as cytosolic mtDNA content from Hep3B cells subjected to siRNA knockdown of MxB and/or OPA1 (Fig. 5m, n). Interestingly, total DNA levels are nearly doubled in the knockdown cells compared with control cells. Consistent with the morphological measurements, cytoplasmic mtDNA levels in OPA1-knockdown cells are modestly increased compared with control cells, whereas these levels are markedly increased (three to four fold) in MxB-knockdown cells. To test whether the MxB-induced loss of mtDNA is dependent upon OPA1 function, Hep3B cells treated with siRNAs to both OPA1 and MxB are subjected to morphometry and qPCR as described above. When both of these dynamin family members are reduced, the loss of mtDNA into the cytoplasm trends toward control levels, suggesting that OPA1 may play a role in the genomic instability induced by a reduction of the MxB protein.

## Discussion

In this study, we report an unexpected subcellular distribution and function for the dynamin family member MxB at the mitochondria. Although MxB is well described to exhibit an IFN-inducible expression^20,47^, we find it is also expressed at basal levels in several untreated tissues including human liver, lymph node, testis, and tonsil, as well as pig liver (Fig. 1e). Further, MxB is expressed in a variety of hepatocyte cells including primary hepatocytes (Fig. 1f) and this expression is markedly increased upon IFN treatment (Fig. 1g). This basal level of expression may suggest that MxB performs housekeeping functions in cells in addition to its anti-viral action. Although HeLa cells do not show an MxB band in the blot provided, this can be resolved upon higher protein loading and/or longer exposure of the blot. In addition, alterations of MxB function in these cells lead to disrupted mitochondria (Fig. 1 and Supplementary Fig. 2). Further, despite our continued efforts, we have been unable to isolate viable MxB-knockout clones using CRISPR/Cas9 in HeLa or Hep3B cells, suggesting a critical function of this protein. The higher molecular weight band of MxB in human liver (Fig. 1e), Hep3B, HepG2, and human primary hepatocytes (Fig. 1f) showed prominent expression. Interestingly, when treating cells with IFN, the short form of MxB is induced, indicating that it may exist constitutively with the long form of MxB in the liver/hepatocytes. However, this lower molecular weight form of MxB needs to be confirmed to indeed be a truncated form of MxB.

The widely reported distribution of MxB at the cytoplasmic face of nuclear pores is consistent with this protein containing an N-terminal nuclear localization sequence (NLS)^34^, making it readily available to prevent the nuclear uptake and chromosome integration of lentiviruses such as HIV-1^23^ into the host genome. In addition to the reported localization at nuclear pores, numerous other studies have described a cytoplasmic distribution for MxB, which we believe may have represented the mitochondria. From the data described above, the localization of MxB at the mitochondria is observed consistently in several different cell types, while using multiple MxB antibodies or tagged constructs (Fig. 1a–d and Supplementary Fig. 1a, b). Although all of these reagents indicate a nuclear and mitochondrial distribution for MxB, distinctions in the types of mitochondrial staining were observed, which varied from evenly distributed to small or large puncta. Digital recording of live Hep3B cells expressing MxB-mCherry demonstrated that a single cell may display all of these distributions (Fig. 2). It was particularly surprising that such large aggregates of MxB can form and dissociate, as static images of these structures suggested the formation of insoluble or denatured aggregates of a tagged protein. The dissolution of these aggregates suggests otherwise and may provide insights into the function of this antiviral dynamin protein. Whether this cycling process leads to an association of MxB with the surface or interior of the mitochondria needs to be determined. We do observe that the mitochondrial distribution is significantly increased in cells expressing an MxB K131A mutant (Fig. 4h, i), a finding that provided us with the initial motivation for pursuing the mitochondrial association.

In addition to the observed fluorescence localization of MxB to the mitochondria, substantial amounts of MxB were found in isolated organelle fractions from pig liver that coincided with mitochondrial enrichment as assessed by CoxIV levels (Fig. 4a). Equally supportive are the findings from the organelle protein protection assays, suggesting that, as observed for OPA1 (Fig. 4b)^40,48^, MxB is protected from mild trypsin exposure of 0.2 mg ml^−1^ and it may reside within the inner mitochondrial membrane. In contrast to OPA1, MxB remains resistant to proteolysis at higher trypsin concentrations, as is the case for CoxIV, suggesting MxB may indeed reside on the inner membrane or in the matrix within a protective complex. The mitochondrial localization of WT MxB is less clear at the ultrastructural level using the APEX2-EM method, as both the inner and outer mitochondrial membranes appeared darker than observed in control untransfected cells (Fig. 4c–e and Supplementary Fig. 4). It is unclear, however, if this indicates that MxB resides on both the inner and outer membranes or the WT protein has a more modest, and perhaps transient, interaction with the inner membrane. A pronounced mitochondrial staining was observed in cells expressing an APEX2-tagged MxB K131A mutant protein. In these cells, the reaction product appeared to fill the matrix in its entirety with the most intense staining coating the cristae membranes while leaving, what we interpret to be, the intermembrane space unstained. This enhanced association of the GTPase mutant was also observed in transfected cells viewed by fluorescence microscopy (Fig. 4h, i). It will be important to define how MxB is targeted to the mitochondria as opposed to the nuclear envelope and how these localizations are exclusive to MxB compared with MxA. Defining this targeting process will further strengthen the concept of MxB as a bona fide mitochondrial dynamin family member, while providing new insights into its function.

GTPase defective mutants in the conventional dynamins are generally believed to bind GTP, at perhaps a reduced affinity, but remain in a bound state as hydrolysis is defective^49–51^. GTP-bound dynamin is thus more likely to favor polymer assembly while associating with membranes in cells^52^, a state also induced by the GTP analog GTPγS as observed using in vitro assays^53,54^. Thus, the analogous GTPase mutation in MxB could explain the marked increase in mitochondrial association and represent a key functional step during its GTP binding/hydrolysis cycle. In contrast to conventional dynamins, recent reports have suggested that GTP binding disassembles both MxB and MxA polymers^28,55,56^. As these dynamin-related family members are largely assumed to interact with virally derived proteins, the membrane affinity for the MxB K131A or MxA K83A GTPase-defective mutants appears to be understudied.

The distribution of MxB reported in this study suggests that it likely has multiple functions in cells beyond that of restricting a single family of infectious virus. MxA has been implicated in attenuating the infection of several distinct virus families with either RNA or DNA genomes at multiple cellular sites that include the nucleus as well as the smooth ER^25^. This is likely to be the case for MxB, as an evolutionary analysis suggests that MxB plays a central and conserved role in the IFN response to a broader range of pathogens than is currently appreciated^57^. The very recent observation that MxB acts as a potent pan-herpes virus restriction factor^58^ supports this premise and may represent the first of many other MxB sensitive viral pathways yet to be identified.

As additional insights into the functions of the Mx proteins expand, it is likely that newly identified intracellular localizations will also increase. Conventional dynamins have been reported to participate in a wide variety of key cellular processes beyond the liberation of caveolae or clathrin-coated pits from the plasma membrane. Some of these functions include budding of nascent secretory vesicles from the trans-Golgi network, vesiculation of autophagosomes and lysosomes, as well as centriole separation and midbody scission during mitosis^5,6,50^. MxB has been reported to possess a NLS at the N-terminus and is likely to be expressed in at least two forms^34^. It is attractive to predict that the form lacking the N-terminal NLS is targeted to the mitochondria, although our expression studies have not supported this concept. It is also not clear whether the basally expressed form is more or less prone to associate with the nucleus or mitochondria compared with the IFN-induced form.

The fact that alterations in MxB levels have a pronounced effect on mitochondrial shape and integrity is consistent with the fact that this protein appears to have a mitochondrial distribution. As displayed in Fig. 3 and Supplementary Fig. 2, manipulation of MxB levels in cells by either siRNA or shRNA knockdown or overexpression of WT or mutant MxB protein leads to distortions in mitochondrial shape along with a profound disruption in the organization of mitochondrial cristae. These changes accompany a marked loss of mitochondrial function such as an inability to maintain a proton gradient (Fig. 5a–f) and a substantial translocation of the mitochondrial nucleoids (mtDNA) into the cytoplasm (Fig. 5g–n). We were surprised to find that in addition to the expulsion of the nucleoids from the mitochondria into the cytoplasm in MxB-knockdown cells, these cells also appeared to possess an increase in the total mtDNA content as assessed by qPCR. This was observed repeatedly in cells treated with siRNA to either MxB or OPA1. Consistent with this finding is a study of muscle fibers from patients with OPA1 mutations, showing a two- to fourfold increase in mtDNA copy number^59^. The authors suggest this increase represents a compensatory mitochondrial proliferative response to maintain a needed level of WT mtDNA.

The stability of the mitochondrial genome appears to be directly related to the integrity of mitochondrial cristae, as has been suggested for the yeast protein MGM1, named after its involvement with mitochondrial genome maintenance^60^. A putative role for MxB in cristae fusion or genome tethering based on its localization and effects on mitochondrial structure is certainly less developed than what is now known about the yeast MGM1 and mammalian OPA1 proteins after many years of investigations^16,61,62^. Future detailed studies on the role of MxB on the inner mitochondrial membrane dynamics will be important, although challenging, as defining the functions of putative inner mitochondrial membrane proteins are particularly difficult. It is well documented that shape, as well as cristae number and structure, are often altered in response to disease, energy burden or metabolic state of the host cells^63,64^. Indeed, since the discovery of yeast DNM1^65^ and the mammalian homolog DRP1/DLP1^66^ almost two decades ago, its precise role in mitochondrial fission remains somewhat elusive, particularly with the observation that conventional Dyn2 is recruited to the constriction site and may provide the final scission event^18^.

From our perspective, what makes the findings of this current study so exciting is the concept of a dynamin family member that is markedly induced in response to viral infection also participates in mitochondrial form, function, and integrity. Further, the two known viruses which MxB attenuates are known to utilize or modify mitochondria during infection. HIV-1 infection is well described to cause alterations in mtDNA and induce mitochondria-centric apoptosis^67,68^. In addition, herpes virus, a recently identified MxB target^58^, was originally observed to have an intimate association with the host’s cell mitochondria over 60 years ago^69^. It is now well known that infection by this virus results in a rapid elimination of mtDNA from the host cells^70,71^. Thus, it is especially attractive to postulate that MxB could be inducing genomic changes in the host’s cell mitochondria as part of the innate immune response. It is now clear that the inappropriate discharge of mtDNA into the cytoplasm is recognized by the host cell as a “prokaryotic infection event” that engages the DNA sensor cGAS to promote STING-dependent signaling^45,72,73^. It will be particularly interesting to test whether MxB is responsible for the observed mitochondrial release of mtDNA during infection by herpes virus and other pathogens.

## Methods

### Antibodies and reagents

Rabbits were immunized with the keyhole limpet hemocyanin-conjugated human MxB N-terminal peptide, 5′-KPWPYRRRSQFSSRKYLKKEMNSFQQQP-3′, and the crude antisera were affinity purified using an agarose column conjugated with the appropriate high-performance liquid chromatography-purified synthetic peptide and a low pH elution buffer, according to the manufacturer’s directions (Pierce Chemical Co., Rockford, IL). The purified anti-MxB antibody (immunofluorescence staining (IF) 5 μg ml^–1^; WB 1:1000) was dialyzed (molecular weight cut-off 12,000–14,000 kDa) against Dulbecco’s phosphte-buffered saline (PBS) (D-PBS; 8.1 mM Na_2_HPO_4_, 1.2 mM KH_2_PO_4_ pH 7.2, 138 mM NaCI, 2.7 mM KCI, 0.9 mM CaCI_2_, 0.5 mM MgCl_2_) containing 0.04% NaN_3_, concentrated using polyethylene glycol, and in some cases, 2 μg ml^–1^ bovine serum albumin (BSA) was added^74^. The other anti-MxB antibodies [Guinea Pig (IF 1:200; WB 1:1000) and Rabbit (IF 5 μg ml^–1^; WB 1:500)] were a generous gift from Dr Ilkka Julkunen^34^ and Dr Chen Liang^23^. The anti-MX2 rabbit polyclonal antibody (NBP1-81018) (IF 1:100; WB 1:500) was from Novus (Centennial, CO). The CoxIV (3E11) (IF 1:250; WB1:1000) rabbit mAb and (4D11-B3-E8) (IF 1:100; WB1:1000) mouse mAb; Tom20 (D8T4N); (WB1:1000) rabbit mAb; OPA1 (D6U6N) (WB1:1000) rabbit mAb; GM130 (D6B1) XP (WB1:1000) rabbit mAb; and GAPDH (D16H11) XP (WB1:1000) rabbit mAb were from Cell Signaling (Danvers, MA). The OPA1 mouse mAb (WB1:500) was from BD Biosciences (San Jose, CA). The mtDNA antibody (WB 1:100) was from EMD Millipore (Temecula, CA). The calnexin-ER marker (WB 1:1000) was from Abcam (Cambridge, MA). The anti-Actin (WB 1:2000) was from Sigma (St Louis, MO). Goat anti-rabbit and goat anti-mouse secondary antibodies conjugated to either Alexa-Fluor-488 or -594 used for IF staining (1:500) were all obtained from Thermo Fisher Scientific (Rockford, IL), and horseradish peroxidase-conjugated goat anti-rabbit and goat anti-mouse were from BioSource International, Inc. (Camarillo, CA), which were used for WB analysis. Miniprep Express^TM^ Matrix was from MP Biomedicals (Solon, OH). Restriction enzymes were from New England Biolabs (Ipswich, MA). DNA ladders (1 kb and 1 kb plus) were from Invitrogen (Carlsbad, CA) and all other chemicals and reagents, unless otherwise stated, were from Sigma (St Louis, MO). Unprocessed blottings of MxB can be found in Supplementary Fig. 4.

### Plasmid construction

The following primers were used to amplify MxB WT-pCR3.1 (without tag) from the plasmid pBS-T7/MxB, a generous gift from Dr G Kochs: MxBWT5′-ATG TCTAAGGCCCACAAGCCTTGGCCCT-3′; MxBWT3′-TCAGTGGATCTCTTTGCT GGAG-3′. Full-length PCRs were performed using the XL PCR kit (Applied Biosystems, Branchburg, NJ) and the PCR fragments were ligated into the eukaryotic expression TA-vector pCR3.1 (Invitrogen, Carlsbad, CA). The MxB inserts from the pCR3.1 constructs were excised by digestion with the corresponding enzymes (XhoI and BamHI for GFP and mCherry tag; BamHI and XhoI for Myc tag) and sub-cloned into the expression vectors pEGFP-N1/pmCherry-N1 (Clontech, Palo Alto, CA) and pcDNA3.1 (Invitrogen, Carlsbad, CA). The constructs inserted into pEGFP-N1 /pmCherry-N1 and pcDNA3.1 have no intervening stop codons. MxB K131A was generated using PCR-based mutagenesis methods. To construct MxB WT/K131A-APEX2-pCR3.1, we purchased APEX2-OMM^41^ plasmid from Addgene (Cambridge, MA) and reconstructed APEX2 into MxB WT/K131A-pCR3.1. The mito-GFP^37^ was made using the MTS (mitochondrial-targeting sequence) from IVD (Isovaleryl CoA dehydrogenase) for the template and ligated into pEGFP N1 vector by EcoRI and BamHI cut sites. All plasmids were purified with the plasmid Maxi kit from Qiagen (Germantown, MD) and DNA constructs were verified by restriction enzyme analysis and sequencing (The Mayo Molecular Biology Core; GENEWIZ, South Plainfield, NJ). Sequences were analyzed using DNA* analysis software (DNA star, Madison, WI).

### siRNA/shRNA and transfections

siRNAs targeting Human Mx2 (M-011736-01-0005) and OPA1 (M-005273-00-0005) were purchased from Dharmacon (Lafayette, CO) and transfected with Lipofectamine RNAiMAX (Invitrogen, Carlsbad, CA) following the standard protocol. Mx2 shRNA and TRC vector (shRNA control) were purchased from Sigma (St Louis, MO) and delivered into cells following the Lentiviral particles standard protocol (Santa Cruz).

### Tissue, cell culture, and transfection

Human tissue was from the Mayo Clinic Biobank for Gastrointestinal Health Research (Institutional Review Board (IRB) 622-00) and was de-identified frozen tissue. Consent and approval were waived, because the IRB determined the frozen tissue did not constitute research involving human subjects as defined under 45 CFR 46.102. Primary pig hepatocytes isolated and cultured from female, Large Domestic Cross-bred White Pigs, 2–3 months old^74^, were a kind gift from Dr Scott Nyberg, Mayo Clinic. All animals received humane care and procedures were performed with approval of the Institutional Animal Care and Use Committee at Mayo Clinic and are in accordance with guidelines set forth by the National Institutes of Health. Human hepatocytes were from Bioivt (Westbury, NY). HeLa cells, an adenocarcinoma from human cervix (ATCC CCL-2), and Huh-7, a hepatocellular carcinoma from human liver^75^, were grown in Dulbecco’s modified Eagle’s medium, plus 10% fetal bovine serum, 100 U ml^−1^ penicillin, and 100 μg ml^−1^ streptomycin (Gibco, Waltham, MA). Hep3B and HepG2 cells, both hepatocellular carcinoma from human liver (ATCC HB-8064) and (ATCC HB-8065), respectively, were maintained in minimum Eagle’s medium with 10% fetal bovine serum, 100 U ml^−1^ penicillin, and 100 μg ml^−1^ streptomycin (Gibco, Waltham, MA). THP-1 cells are monocyte from acute leukemia (ATCC TIB-202). All cells are cultured in 5% CO_2_ and 95% air at 37 °C incubator. Cells were cultured in T-75 flasks (Fisher Scientific, Pittsburgh, PA). Cells were transfected using Lipofectamine 2000 (Invitrogen, Carlsbad, CA) according to the manufacturer’s protocol.

### Immunofluorescence and electron microscopy

For IF microscopy, cells on coverslips were rinsed with D-PBS and fixed for 20 min with 2.5% formaldehyde in PIPES buffer (0.1 M Pipes pH 6.95, 3 mM MgSO_4_, 1 mM EGTA). After rinsing with D-PBS, cells were permeabilized with 0.1% Triton X-100 in D-PBS for 2 min, rinsed in D-PBS, and incubated in blocking buffer (5% normal goat serum and 5% glycerol in D-PBS) for 1 h at 37 °C. Cells were incubated in primary antibodies diluted in blocking buffer and rinsed repeatedly in D-PBS before incubating in the appropriate fluorescently labeled secondary antibodies diluted in blocking buffer. Cells were then washed extensively with D-PBS, rinsed with distilled water, and mounted on a glass slide with mounting reagent Prolong gold (Invitrogen, Carlsbad, CA). Fluorescence micrographs were acquired using an AxioObserver D.1 epifluorescence microscope (Carl Zeiss, Thornwood, NY) equipped with a 100 W mercury lamp using a ×63, 1.4 NA objective lens, an Orca II digital camera (Hamamatsu Photonics, Hamamatsu, Japan) and ZEN software (Carl Zeiss Microscopy LLC, Thornwood, NY). Images were processed using Adobe Photoshop (Adobe Systems Incorporated, San Jose, CA).

For standard transmission EM, cells on carbon-coated coverslips were rinsed in 37 °C Hank’s buffered salt solution and fixed with 37 °C primary fixative (100 mM cacodylate pH 7.4, 60 mM sucrose, 2.5% glutaraldehyde) for 1 h at room temperature, rinsed three times with washing buffer (100 mM cacodylate pH 7.4, 200 mM sucrose) then fixed in the secondary fixative (50 mM cacodylate pH 7.4, 100 mM sucrose, 1% OsO_4_) for 1 h at room temperature, and rinsed three times in water then fixed in 1% uranyl acetate in water for 1 h at room temperature. Samples were then dehydrated in a graded ethanol series, embedded in Quetol 651 (Ted Pella, Redding, CA), and polymerized in a 65 °C oven overnight. After removal from the oven, the coverslip was removed from the bottom of the sample, the block trimmed down to a trapezoid 1 mm wide at the base, 100 nm thin sections were cut and viewed on a Joel 1200 transmission electron microscope (Jeol Ltd, Tokyo, Japan).

For cells expressing the APEX2 tag, after primary fixation for 30 min, an electron-dense reaction product was generated by incubating the samples with 1 mg ml^−1^ DAB-HCl (3,3’-diaminobenzidine tetrahydrochloride; Electron Microscopy Sciences, Hatfield, PA) + 0.012% H_2_O_2_ in washing buffer for 3 or 6 min at room temperature, washed four times in washing buffer, then processed the same as the other EM samples (secondary fix through embedding).

### Live-cell imaging

Hep3B cells were transfected with MxB-mCherry and mito-GFP in a six-well plate. After transfection, cells were re-plated into 35 mm glass-bottomed imaging dishes (Cell E&G, San Diego, CA) in MEM media with 10% fetal bovine serum, 100 U ml^−1^ penicillin, and 100 μg ml^−1^ streptomycin (Gibco, Waltham, MA). The 35 mm imaging dish was placed in a stage top incubator at 37 °C and 5% CO_2_ on a Zeiss AxioObserver microscope (Carl Zeiss, Thornwood, NY) equipped with a Colibri 7 LED light source, a Zeiss Axiocam 702 digital camera (Carl Zeiss, Thornwood, NY) and images were captured every 5 min for 16–24 h with Zen software (Carl Zeiss, Thornwood, NY).

### Quantitative methods and statistical analysis

For optical measurements of mitochondrial DNA, fluorescence micrographs of Hep3B cells stained for CoxIV and mtDNA were analyzed with ImageJ software^76^ and the degree of overlap was calculated using the Just Another Colocalization Plugin^77^. Briefly, nuclei were deleted in the mtDNA channel, then using the plugin, thresholds were set for the individual channels and the amount of overlap between the two channels was calculated using the Manders M2 coefficient (fraction of mtDNA overlapping with the mitochondria).

For statistical analysis, graphs were generated and statistical analyses performed using the two-tailed *t*-test using GraphPad Prism software (San Diego, CA).

### Rhodamine 123 assay

Fluorescence micrographs of Hep3B cells that were knocked down with siRNA or transfected to express either vector (pmCherry-N1), MxB WT–pmCherry-N1, or MxB K131A–pmCherry-N1 for 5 days. On day 5, cells were loaded with 50 nM Rho123 for 30 min, washed 3 times, and incubated at 37 °C for 90 min prior to any imaging. Images of cells were taken in both fluorescein and mCherry channels, and the mitochondrial fluorescence of the transfected cells was quantified and graphed. Vector-expressing cells or NTsi control cells were treated with carbonyl cyanide m-chlorophenylhydrazone (CCCP) to depolarize the mitochondria and their fluorescence intensity set to zero to calibrate the graphs.

### qPCR measurements of mitochondrial DNA

The whole cell and cytosolic DNA was purified by QIAamp DNA Mini Kit (250) LN:154041343 (Qiagen, Valencia, CA). Quantitative real-time PCR was done using Lightcycler 480 system (Roche Life Science, Indianapolis, IN) and analysis was performed using the Roche analysis software. The thermo-cycler conditions were 95 °C for 5 min, 95 °C for 30 s, 60 °C for 30 s, 72 °C for 30 s, totalling 50 cycles. The primer sequences used to amplify human D-loop were: sense 5′-CATCTGGTTCCTACTTCAGGG-3′ and antisense 5′-CCGTGAGTGGTTAATAGG GTG-3′.

### Mitochondrial purification and protease protection assay

Enriched mitochondria (mito) fractions were isolated from pig liver using a modified protocol at 4 °C^39^. Approximately 10 g of pig liver was resuspended in 50 ml of IB-1 buffer (225 mM mannitol, 75 mM sucrose, 0.5% BSA, 0.5 mM EGTA, and 30 mM Tris-HCl pH 7.4) and homogenized 10 or more strokes at 1500 r.p.m. Homogenate was then centrifuged twice at 740 × *g* for 5 min to separate unbroken cells and nuclei (pellet) from the rest of the fractions (supernatant). The latter was further centrifuged at 9000 × *g* for 10 min to separate crude mitochondrial pellet from cytosolic fraction containing microsomes/ER (supernatant). For isolation of the cytosol and ER, the supernatant was centrifuged at 95,000 × *g* for 30 min using a SW41 rotor (Beckman)—the resulting pellet contains ER with cytosol being present in the supernatant. The crude mito pellet was further gently resuspended (not to disrupt the outer mito membrane) in 20 ml of IB-2 (225 mM mannitol, 75 mM sucrose, 0.5% BSA, and 30 mM Tris-HCl pH 7.4) and centrifuged twice at 10,000 × *g* for 10 min. After the final centrifugation, crude mito pellet was resuspended in 2.25 ml of MRB (250 mM mannitol, 5 mM HEPES, and 0.5 mM EGTA). To isolate pure mito fraction, 1.8 ml of crude mito fraction was layered on Percoll gradient followed by centrifugation at 95,000 × *g* for 30 min. A dense band containing purified mito at the bottom of the tube was collected using a glass Pasteur pipette and diluted 10× with MRB followed by two centrifugations at 6300 × *g* for 10 min. The final pellet was resuspended in a small volume of MRB and used for mitochondria protease protection assay^40^. Briefly, 100 μg of freshly isolated pure mito fraction was treated with 0.2 or 1 mg ml^−1^ trypsin in 100 μl of mitochondria digestion buffer (10 mM sucrose, 0.1 mM EGTA, and 10 mM Tris/HCl pH 7.4) for 20 min on ice. The reaction was terminated by boiling the samples in Laemmli buffer + β-mercaptoethanol for 5 min.

### Rigor and reproducibility

The type of statistical test, *n*-values, and *P*-values are all listed in the figure legends or in the figures. All microscopy data were quantified using ImageJ or Fiji, and figures were assembled using Adobe Photoshop and are representative of at least three independent experiments with similar results. All experiments were performed at least three times, except for Fig. 4a–b, which was fractionated one time and blotted three times, Supplementary Fig. 2a–c, which was done one time and represents 18 cells, and Supplementary Fig. 2d, i, which was each done one time. Supplementary Movie 1 is representative of 24 cells over 6 experiments and Supplementary Movies 2 and 3 are representative of 33 cells over 5 experiments.

### Reporting summary

Further information on research design is available in the Nature Research Reporting Summary linked to this article.

## Supplementary information

Supplementary Information

Description of Additional Supplementary Files

Supplementary Movie 1

Supplementary Movie 2

Supplementary Movie 3

Reporting Summary

## Data Availability

The datasets generated and analyzed during this study are available from the corresponding author upon reasonable request. Figures 1–5 and Supplemental Figs. 1–5 have associated raw data. Data for the graphs in the manuscript are available in the associated source file.

## Source Data

Source Data
