# Supplementary material for: The anti-viral dynamin family member MxB participates in mitochondrial integrity
Source: Nat Commun. 2020 Feb 26;11:1048. doi: 10.1038/s41467-020-14727-w (PMC7044337; doi:10.1038/s41467-020-14727-w)
Supplement: Supplementary file 1 — Supplementary Information [file 41467_2020_14727_MOESM1_ESM.pdf]

Supplementary information for

The Anti-Viral Dynamin Family Member MxB Participates in Mitochondrial Integrity

Cao et al.

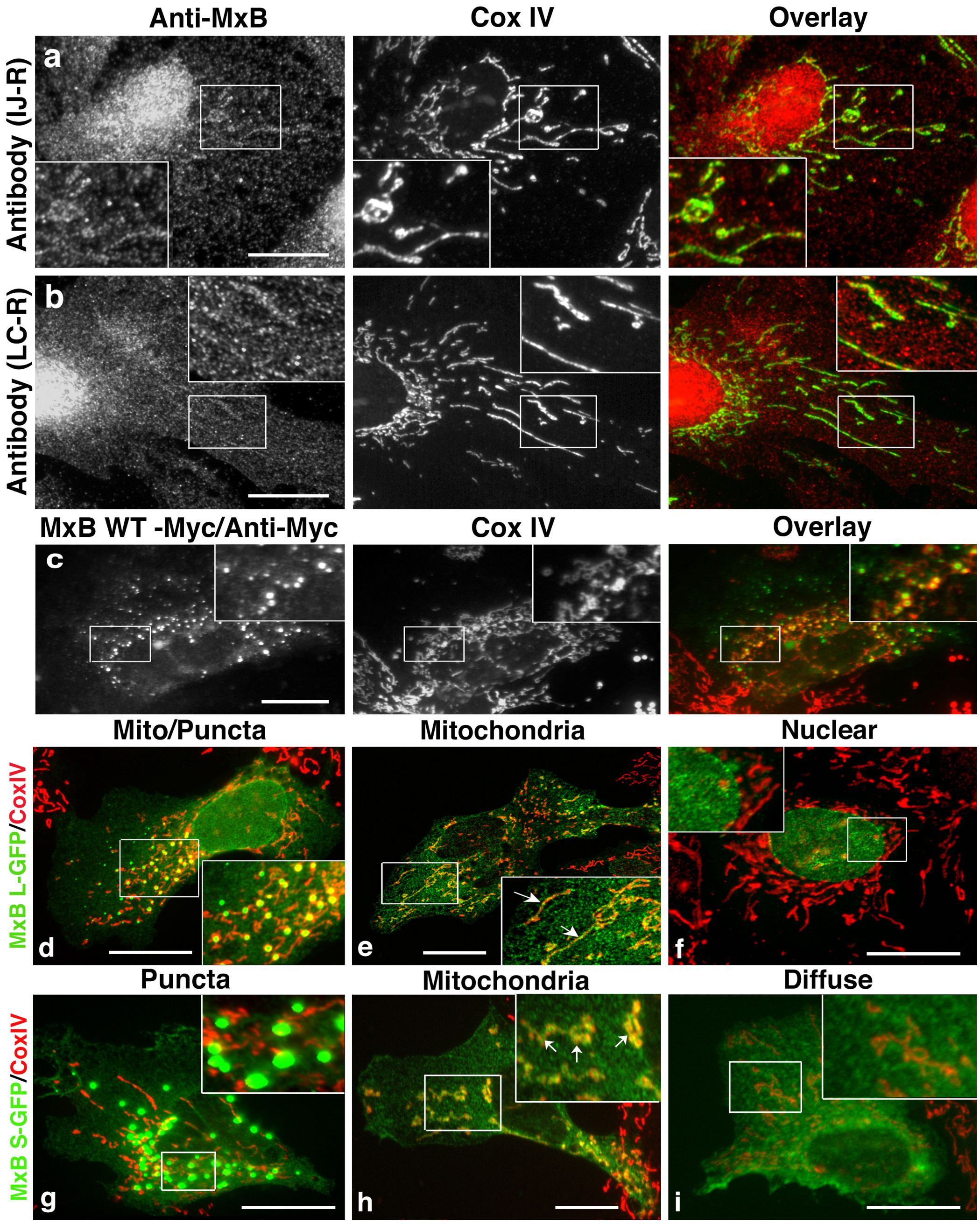

j GFP / Myc tagged MxB L & S form distribution in Hep3B cells (%)

Phenotype / Form	Mito/Puncta	Puncta	Nucleus	Mitochondria	Diffuse
MxB L -GFP	20.8	39.4	11.4	28.4	–
MxB S -GFP	–	54.3	–	15.6	30.1
MxB L -Myc	23.6	43.2	4.7	23.5	–
MxB S -Myc	–	60.9	–	9.2	29.9

Supplementary Figure 1 Mitochondrial localization of MxB using additional antibody reagents and distributions of MxB Long and Short forms. a,b, Hep3B cells were fixed and stained for CoxIV or MxB antibodies from additional sources (see reagents section for source). Both antibodies give punctate fusiform staining that co-localizes with the mitochondrial marker CoxIV. **c**, Hep3B cells

were transfected to express a MxB WT-Myc construct then fixed and stained for Myc and CoxIV. MxB puncta are observed within the CoxIV mitochondrial staining. **d-f**, Hep3B cells transfected to express GFP-tagged MxB Long form construct then fixed and stained for CoxIV. **g-i**, Hep3B cells transfected to express GFP-tagged MxB Short form construct then fixed and stained for CoxIV. **j**, Table for GFP- and Myc-tagged MxB Long and Short form distribution in Hep3B cells from 246 cells from 3 distinct experiments. Scale Bar, 10μm.

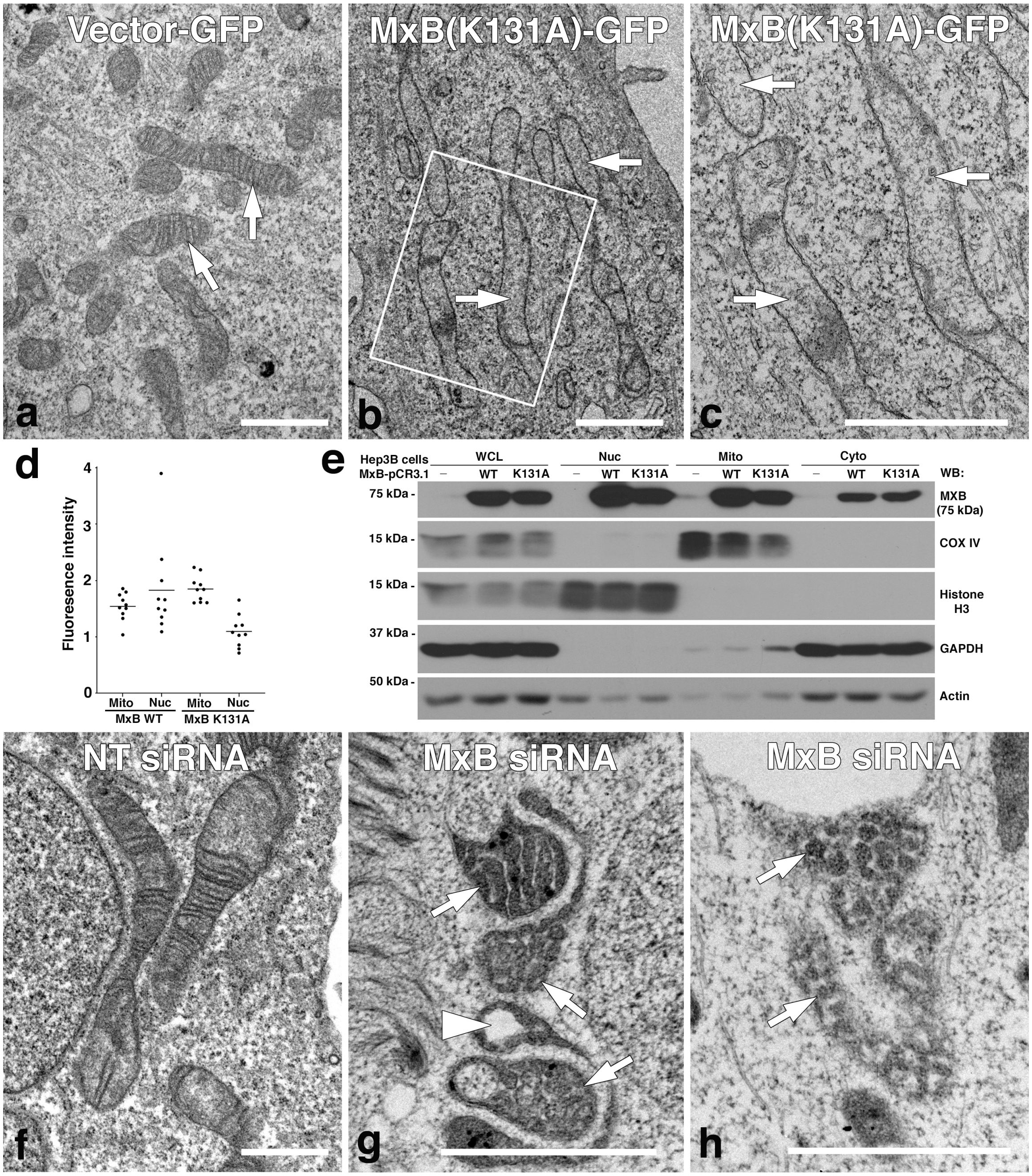

Supplementary Figure 2 Additional electron micrographs, immunofluorescence quantitation and biochemical fractionation of compromised mitochondria in MxB-altered cells. a-c, HeLa cells were transfected for 48 hrs with GFP-vector alone or MxB K131A-GFP prior to fixation and viewing by EM. Normal cristae are observed in the mitochondria of the vector-only control cells compared to that of the cells expressing the mutant protein, which possess largely hollowed organelles with very few residual cristae. Hollow mitochondria were not observed in any control treated cells. **d,** Hep3B cells expressing GFP-tagged MxB WT or K131A constructs were fixed and

quantitated for MxB distribution to mitochondria (Mito) and nucleus (Nuc) by fluorescence microscopy (line indicates mean, N=10 cells/ condition). **e,** MxB WT and K131A (without tags) are expressed in Hep3B cells and analyzed by sub-cellular fractionation. After fractionation assay, all fractions were probed with marker antibodies to mitochondria (CoxIV), nucleus (Histone H3), GAPDH and actin, (WCL=Whole cell lysate). **f-h,** EM of Hep3B cells treated with siRNA to MxB for 5 days prior to fixation. Mitochondria in control-treated cells are larger in Hep3B compared to the HeLa cells and also exhibit numerous cristae. MxB siRNA treated cells have mitochondria which are twisted with vacuoles (arrowheads) while the cristae are either atrophied or vesiculated (arrows). Scale Bars, 1 μm.

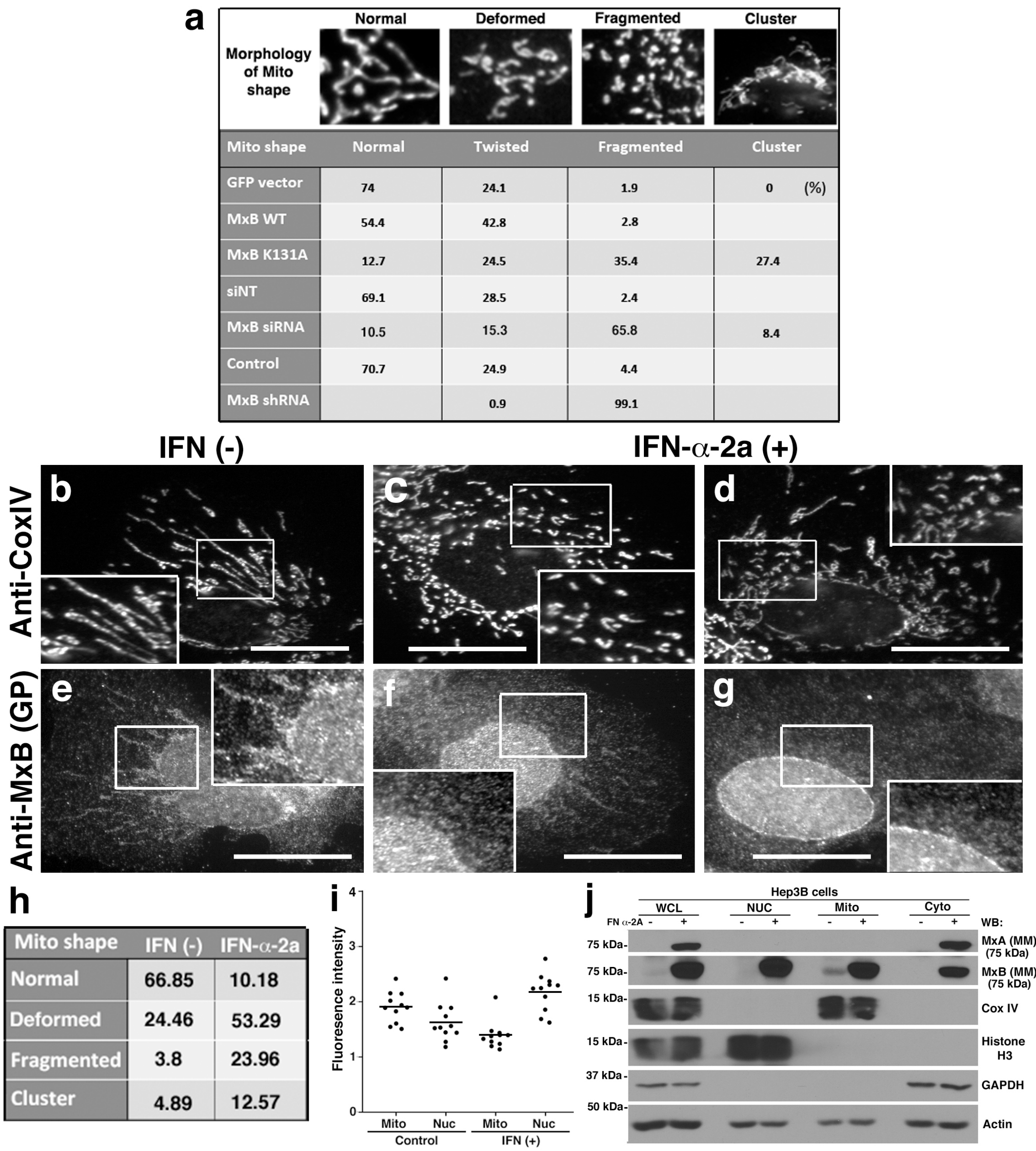

Mito shape	Normal	Twisted	Fragmented	Cluster
GFP vector	74	24.1	1.9	0 (%)
MxB WT	54.4	42.8	2.8	
MxB K131A	12.7	24.5	35.4	27.4
siNT	69.1	28.5	2.4	
MxB siRNA	10.5	15.3	65.8	8.4
Control	70.7	24.9	4.4	
MxB shRNA		0.9	99.1	

Mito shape	IFN (-)	IFN-α-2a
Normal	66.85	10.18
Deformed	24.46	53.29
Fragmented	3.8	23.96
Cluster	4.89	12.57

Supplementary Figure 3 Mitochondrial morphology altered by MxB expression or IFN induced MxB expression. a, Additional table representative of figure 3 (c & l). **b-d,** Fluorescence images of Hep3B cells stained for CoxIV **b**, without, or **c-d,** with IFN treatment. **e-g,** Hep3B cells with or without IFN treatment, stained with anti-MxB (GP) antibody. **h,** The table of mitochondrial shape with or without IFN treatment, from 636 control or MxB transfected cells from 3 distinct experiments. **i,** Quantitation of MxB distribution of fluorescence images of Hep3B cells with or without IFN treatment (line indicates mean, N=11 cells/ condition). **j,** Western blot analysis of MxB distribution observed post IFN treatment. Fractions include: whole cell lysate (WCL), a nuclear pellet (Nuc), a

mitochondria fraction (Mito), a cytosolic fraction (Cyto); all fractions were probed with MxA, MxB and marker antibodies to mitochondria (CoxIV), nucleus (Histone H3), GAPDH and actin.

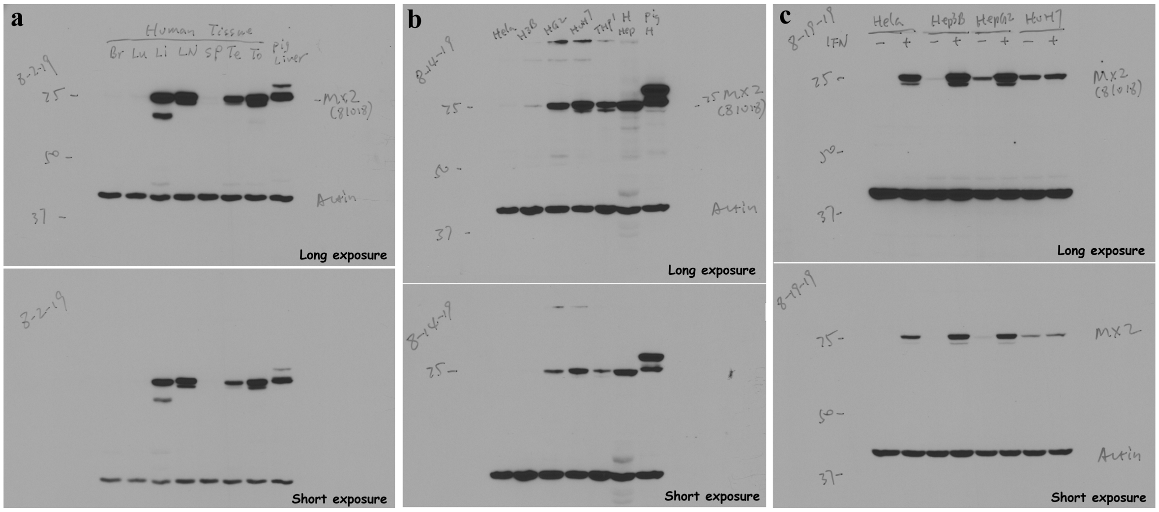

Supplementary Figure 4 Western blots, scans of original uncropped and unprocessed blots used in figure 1e-g. a, The WB is shown in Figure 1e, upper is long exposure, and lower is short exposure. Figure 1e used short exposure of WB.

b, The WB is shown in Figure 1f, upper is long exposure, and lower is short exposure. Figure 1f used long exposure of WB.

c. The WB is shown in Figure 1g, upper is long exposure, and lower is short exposure. Figure 1e used short exposure of WB.

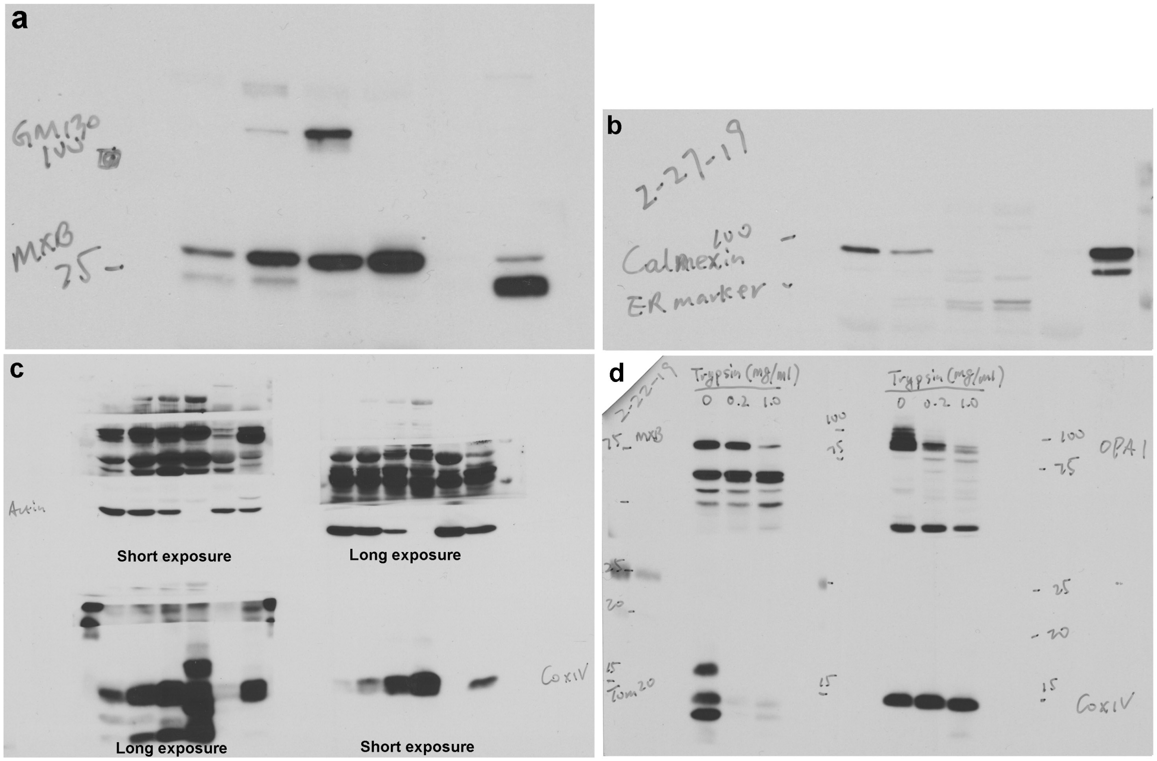

Supplementary Figure 5 Western blots, scans of original uncropped and unprocessed blots used in figure 4a & b. **a**, The WB is shown in Figure 4a, upper blot is Golgi marker GM130, and lower blot is anti-MxB blot. **b**, The WB is shown in Figure 4a, blot with ER marker Calnexin. **c**, The upper and lower bands of WB is shown in Figure 4a that was blotted for actin. In Figure 4a, we used short exposure. Lower blot is for mitochondrial marker CoxIV. In Figure 4a, we used short exposure. **d**, The WB is shown in Figure 4b. Upper left is MxB blot and right is OPA1 blot; Lower left is outer mitochondrial membrane marker Tom20, and right is inner mitochondrial membrane marker CoxIV.
