# Supplementary material for: The anti-viral dynamin family member MxB participates in mitochondrial integrity
Source: Nat Commun. 2020 Feb 26;11:1048. doi: 10.1038/s41467-020-14727-w (PMC7044337; doi:10.1038/s41467-020-14727-w)
Supplement: Supplementary file 2 — Description of Additional Supplementary Files [file 41467_2020_14727_MOESM2_ESM.pdf]

Description of Additional Supplementary Files

File Name: Supplementary Movie 1
Description: A Hep3B cell expressing MxB-mCh imaged one frame every five minutes for over 16 hours 40 minutes. Note the formation of MxB-mCh foci which form and disassemble over the course of the video.

File Name: Supplementary Movie 2
Description: A Hep3B cell expressing MxB-mCh (red) and mito-GFP (white) imaged one frame every five minutes for over 16 hours 40 minutes. The red MxB-mCh spots coalesce in the cytosol where they quickly associate with the mitochondria.

File Name: Supplementary Movie 3
Description: A second example of a Hep3B cell expressing MxB-mCh (red) and mito-GFP (white) imaged one frame every five minutes for over 16 hours 40 minutes. The red MxB-mCh spots coalesce in the cytosol where they quickly associate with the mitochondria.
