# Supplementary material for: The anti-viral dynamin family member MxB participates in mitochondrial integrity
Source: Nat Commun. 2020 Feb 26;11:1048. doi: 10.1038/s41467-020-14727-w (PMC7044337; doi:10.1038/s41467-020-14727-w)
Supplement: Supplementary file 6 — Reporting Summary [file 41467_2020_14727_MOESM6_ESM.pdf]

nature research

Corresponding author(s): Mark McNiven

Last updated by author(s): Oct 31, 2019

Reporting Summary

Nature Research wishes to improve the reproducibility of the work that we publish. This form provides structure for consistency and transparency in reporting. For further information on Nature Research policies, seeAuthors & Referees and theEditorial Policy Checklist .

Statistics

For all statistical analyses, confirm that the following items are present in the figure legend, table legend, main text, or Methods section.

n/a	Confirmed	
☐	☒	The exact sample size (*n*) for each experimental group/condition, given as a discrete number and unit of measurement
☐	☒	A statement on whether measurements were taken from distinct samples or whether the same sample was measured repeatedly
☐	☒	The statistical test(s) used AND whether they are one- or two-sided<br>*Only common tests should be described solely by name; describe more complex techniques in the Methods section.*
☒	☐	A description of all covariates tested
☒	☐	A description of any assumptions or corrections, such as tests of normality and adjustment for multiple comparisons
☐	☒	A full description of the statistical parameters including central tendency (e.g. means) or other basic estimates (e.g. regression coefficient) AND variation (e.g. standard deviation) or associated estimates of uncertainty (e.g. confidence intervals)
☒	☐	For null hypothesis testing, the test statistic (e.g. *F*, *t*, *r*) with confidence intervals, effect sizes, degrees of freedom and *P* value noted<br>*Give P values as exact values whenever suitable.*
☒	☐	For Bayesian analysis, information on the choice of priors and Markov chain Monte Carlo settings
☒	☐	For hierarchical and complex designs, identification of the appropriate level for tests and full reporting of outcomes
☒	☐	Estimates of effect sizes (e.g. Cohen's *d*, Pearson's *r*), indicating how they were calculated

Our web collection on statistics for biologists contains articles on many of the points above.

Software and code

Policy information about availability of computer code

Data collection: Fluorescence microscopy images were acquired using Zen software (Zen blue, version 2.3, Carl Zeiss Microscopy LLC, Thornwood, NY). Electron microscopy images were acquired using Digital Micrograph (version 3.1, Gatan, Inc., Pleasanton, CA). Quantitative real-time PCR was conducted using Lightcycler 480 system (LCS480 version 1.5.0.39, Roche life science, Indianapolis, IN).

Data analysis: Fluorescence co-localization data was analyzed using ImageJ software (version 1.52a, National Institutes of Health, USA) and the JACOP (Just Another Co-localization Plug-in) plug-in. Quantitative real-time PCR data was analyzed using Roche analysis software (LCS480 version 1.5.0.39, Roche life science, Indianapolis, IN).

For manuscripts utilizing custom algorithms or software that are central to the research but not yet described in published literature, software must be made available to editors/reviewers. We strongly encourage code deposition in a community repository (e.g. GitHub). See the Nature Research guidelines for submitting code & software for further information.

Data

Policy information about availability of data

All manuscripts must include a data availability statement. This statement should provide the following information, where applicable:

- Accession codes, unique identifiers, or web links for publicly available datasets
- A list of figures that have associated raw data
- A description of any restrictions on data availability

The data sets generated and analyzed during this study are available from the corresponding author upon reasonable request. Figures 1-5 and supplemental figures 1-3 have associated raw data.

Field-specific reporting

Please select the one below that is the best fit for your research. If you are not sure, read the appropriate sections before making your selection.

☒ Life sciences ☐ Behavioural & social sciences ☐ Ecological, evolutionary & environmental sciences

For a reference copy of the document with all sections, see nature.com/documents/nr-reporting-summary-flat.pdf

Life sciences study design

All studies must disclose on these points even when the disclosure is negative.

Sample size	No sample sizes were pre-determined. For all quantitation, experiments were performed at least 3 times with N>=10
Data exclusions	Samples were excluded from analysis if Western blotting showed poor knockdown in siRNA or shRNA experiments.
Replication	Reproducibility of data was addressed by keeping culturing of cells the same as well as keeping the timing of experiments consistant.
Randomization	No randomization was performed. Samples were allocated to different groups based upon over-expression or knockdown of specific proteins.
Blinding	For computer based quantitation of Rhodamine 123 uptake, images of random fields were acquired, and all samples were analyzed using the same software parameters. Morphological analysis of mitochondria was performed by a qualitative grouping of organelle shapes into 4 broad categories. For mtDNA image quantitation, samples were manually thresholded then mtDNA localization was determined by non bias analysis software.

Reporting for specific materials, systems and methods

We require information from authors about some types of materials, experimental systems and methods used in many studies. Here, indicate whether each material, system or method listed is relevant to your study. If you are not sure if a list item applies to your research, read the appropriate section before selecting a response.

Materials & experimental systems

n/a	Involved in the study	
☐	☒	Antibodies
☐	☒	Eukaryotic cell lines
☒	☐	Palaeontology
☐	☒	Animals and other organisms
☒	☐	Human research participants
☒	☐	Clinical data

Methods

n/a	Involved in the study	
☒	☐	ChIP-seq
☒	☐	Flow cytometry
☒	☐	MRI-based neuroimaging

Antibodies

Antibodies used	Human anti-MxA rabbit polyclonal and human anti-MxB rabbit polyclonal antibodies were developed by the McNiven lab, other anti-MxB (Guinea Pig and Rabbit) antibodies were a generous gift from Dr. Ilkka Julkunen and Dr. Chen Liang, respectively. The anti-MX2 rabbit polyclonal antibody (NBP1-81018) was from Novus (Centennial, CO). The anti-CoxIV (3E11) rabbit mAb and (4D11-B3-E8) mouse mAb; anti-Tom20 (D8T4N) rabbit mAb; anti-OPA1 (D6U6N) rabbit mAb; anti-GM130 (D6B1) XP rabbit mAb; anti-GAPDH (D16H11) XP rabbit mAb were from Cell Signaling (Danvers, MA). The anti-OPA1 mouse mAb was from BD Biosciences (San Jose, CA). The anti-mtDNA antibody was from EMD Millipore (Temecula, CA). The anti-calnexin antibody was from Abcam (Cambridge, MA). The anti-Actin antibody was from Sigma (St. Louis, MO).<br>Goat anti-rabbit and goat anti-mouse secondary antibodies conjugated to either Alexa-Fluor-488 or -594 used for immunofluorescence staining were all obtained from Thermo Fisher Scientific (Rockford, IL), and HRP-conjugated goat anti-rabbit and goat anti-mouse antibodies for Western blot analysis were from BioSource International, Inc. (Camarillo, CA).
Validation	Commercial antibodies were used per manufacturers recommendations and additionally validated by Western blotting of over-expression or knockdown samples. Rabbit polyclonal antibodies made by McNiven lab were purified on a peptide column and validated by Western blot against pre-immune serum, and over-expression or knockdown samples.

Eukaryotic cell lines

Policy information about cell lines

Cell line source(s)	HeLa, Hep3B (Hep 3B 2.1-7), and HepG2 cells were purchased from ATCC. Huhep were from Bioivt (Westbury, NY), Huh-7 cells were kindly provided by Dr. Gregory Gores, THP-1 monocytes were a kind gift from Dr. Daniel Billadeau, Pig hepatocytes were freshly isolated and kindly provided by the Dr. Scott Nyberg lab at Mayo Clinic.
Authentication	HeLa, Hep3B (Hep 3B 2.1-7), and HepG2 cells were purchased from ATCC and Huhep were purchased from Bioivt (Westbury, NY) so no additional authentication was done. Pig hepatocytes were freshly isolated so no additional authentication was done. Huh7 and THP-1 cells were not authenticated.
Mycoplasma contamination	Cells were routinely stained with DAPI and no mycoplasma was detected by DAPI stain.
Commonly misidentified lines (See ICLAC register)	n/a

Animals and other organisms

Policy information about studies involving animals; ARRIVE guidelines recommended for reporting animal research

Laboratory animals	female Large Domestic Cross-bred White Pigs, 2-3 months old.
Wild animals	n/a
Field-collected samples	n/a
Ethics oversight	All animals received humane care and procedures were performed under the guidelines set forth by the Institutional Animal Care and Use Committee at Mayo Clinic and are in accordance with those set forth by the National Institutes of Health.

Note that full information on the approval of the study protocol must also be provided in the manuscript.
